# International beef trade: A value proposition

**DOI:** 10.1093/af/vfy013

**Published:** 2018-06-29

**Authors:** KathrynAnn H Fields, Dustin A Therrien, Dan Halstrom, Joel Haggard, Paul Clayton

**Affiliations:** 1 Department of Public Service and Administration, Bush School of Government and Public Service, Texas A&M University, College Station, TX; 2 Department of Animal Science, College of Agriculture and Life Sciences, Texas A&M University, College Station, TX; 3 U.S. Meat Export Federation, Denver, CO

**Keywords:** U.S. beef, U.S. pork, Agricultural exports, International trade

**Implications**

Over the 9-yr span from 2008 to 2017, the U.S. beef industry increased its revenue by 15% through exports to the Japanese market.
China and Africa are new emerging markets with potential for increased beef marketing opportunities.
Use of hormonal growth promotants and beta adrenergic agonists, and the absence of traceability creates barriers to international trade.

## Introduction

Agricultural trade supports U.S. jobs, encourages investment, and promotes economic growth. Roughly more than 20% of the U.S. agricultural production is exported, making the United States the world’s top exporter of food and agricultural products. Agriculture exports generate 8,000 jobs for every $1 billion in farm exports (USDA-ERS, 2018). International trade is a vital aspect of America’s cattle industry as meat accounts for one of the most significant segments of the U.S. agricultural economy. Beef exports increased in 2017 during a record-breaking year for red meat exports. Exports totaled 1.26 million metric tons, the fourth largest volume on record, and the second largest of the post-bovine spongiform encephalopathy (**BSE**) era, with beef export values reaching a record $7.27 billion, a 15% increase from 2016 (U.S. Meat Export Federation, 2017b).

**Figure 1.**
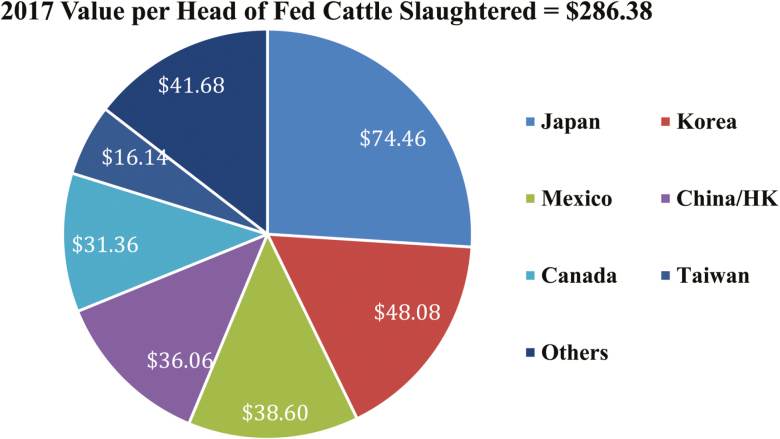
Value per head of fed cattle slaughtered (Halstrom, 2018).

The greatest importers of U.S. beef are Japan, Mexico, South Korea, Hong Kong, Canada, and the Middle East (Halstrom, 2018). Beef export value averaged $286.38 per head of fed slaughter in 2017, up 9% from 2016 (Figure 1). Japan leads importers at $74.46 per head of fed cattle slaughtered, followed by Korea at $48.08 and Mexico at $38.60 (Halstrom, 2018). The U.S. industry is currently marketing a wide range of beef cuts in Japan, including tongues which add $12.50 value per head and short-plates which average $27.00 in additional carcass value.

The U.S. beef industry competes with Canada, Australia, New Zealand, Brazil, Argentina, and Uruguay for the export market. The United States is competitively advantaged because the industry is well known for its quality, safety, and well-designed infrastructure. However, leading competitors, as well as European Union countries, have strong traceability programs in place for food and livestock, which the United States has resisted. The United States faces several additional trade barriers for exporting beef in the global market including high tariffs, restrictive quotas, beta-agonist and hormone residue bans, age restrictions, strict beef cut procedures, and traceability requirements. Although significant trade barriers exist, ample opportunities are present as beef consumption rapidly grows and creates room for global expansion of the beef industry.

## Trade Barriers

The United States has free trade agreements in effect with 20 countries (United States Trade Representative, 2018). Some of these agreements, such as the North American Free Trade Agreement are multilateral agreements among more than one country. Trade agreements have created opportunities for U.S. beef by reducing barriers to exports. Trade agreements reduce and eliminate tariffs and quotas, but also address the barriers that obstruct the flows of goods and services between parties. A tariff is a tax levied on goods transported from one customs areas to another while a tariff rate quota is a quantity limit on imports (Meat and Livestock Australia, 2018).

**Figure 2.**
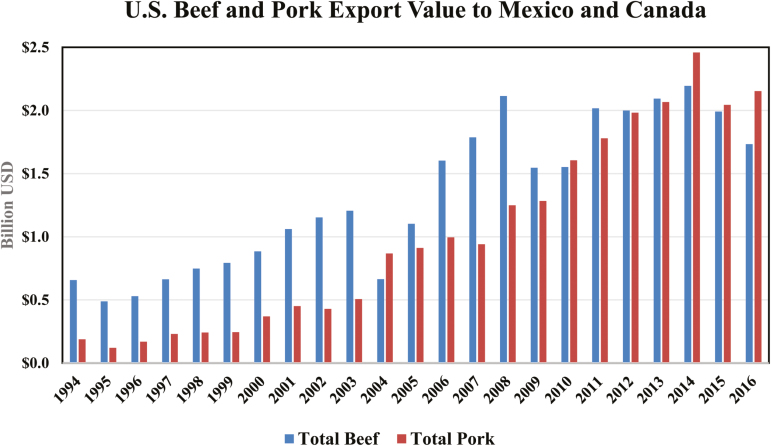
U.S. Beef and Pork Export Value to Mexico and Canada (Halstrom, 2018).

Under the North American Free Trade Agreement, the United States meat exports have zero-tariff market access to both Mexico and Canada. In 2017, beef exports to Mexico and Canada were valued at $1.8 billion or 25% of the total beef market (Figure 2). The United States would face a 20% duty on chilled beef and 25% duty on frozen beef to Mexico without North American Free Trade Agreement (Halstrom, 2018). Since the agreement between the United States, Mexico, and Canada was enacted, food and agricultural products exports have grown dramatically from $11 billion in 1993 to more than $43 billion in 2016 (North American Meat Institute, 2016).

Following the detection of a case of BSE in 2003, many markets closed to U.S. beef for BSE-related reasons, but shortly after, many markets re-opened, at least partially. China had been the most significant consumer of imported beef until the 2003 ban took effect. Japan, one of the largest beef-importing countries, banned the imports of U.S. beef following the case of BSE. In 2005, Japan resumed some imports of U.S. beef from cattle no more than 20 mo of age. By 2013, the increase of cattle aged up to 30 mo has allowed the United States more market access. (Giamalva et al., 2013).

**U.S. Pork in the Chinese Market**

U.S pork has continually increased its presence in the Chinese market over the past decade. Although there have been many challenges, the market is diversifying and provides many opportunities for a wide variety of products.

China was the third largest pork market for the US during the 2013–2015 period, and over the past two years, became the second largest pork market as total imports surged. From 2013 through 2017 the U.S exported over 1.4 million metric tons of pork to China amounting to nearly $3 billion in value. About 55% of the products exported were variety meats and byproducts including pig feet, ears, stomachs, neckbones and other items. The remaining 45% were muscle cuts including hams, shoulders, spare ribs and bellies, among other items. All variety meats are exported frozen and generally, enter wholesale markets where they are distributed through several market layers, eventually reaching consumers in wet markets and small restaurants. Muscle meat items may be exported frozen or chilled, but the cold chain is such that most trade to date has consisted of frozen products. In either case, muscle cut items can be used for further processing, retail and food service.

According to official Chinese government production and trade statistics, China is the world’s largest consumer of pork on a total volume basis, and has per capita consumption of 39.7 kg (87.5 lbs). China has a self-sufficiency rate of 96%. With a 16% market share, share, the US competes with other suppliers including the EU (62% market share), Canada (11%), Chile (2%) and Brazil (7%). In 2013 to 2014 the EU was unable to export to Russia and began selling large amounts of both muscle cuts and variety meats to China for the processed meat sector. During the early spring of 2018, China live hog and pork prices dropped over 30% due to increasing supplies, resulting in lower purchases from all overseas suppliers.

Many market access challenges remain for US exporters. Most recently, actions by the US to impose import duties on steel, aluminum, washing machines and other products resulted in retaliation by China which included the implementation of an additional 25% duty on US pork imports and a return to 100% port inspections.

Although many detailed requirements exist for export to China, U.S. exporters are now accustomed and are generally able to manage the details. Exporting requirements are listed in the USDA FSIS Export Library. All U.S plants are eligible for export but a few are currently de-listed due to various port violations. Bilingual labels are required on each carton including a bilingual insert for each box. All products are subject to port testing for antibiotics, pathogens, and other banned substances. In addition, ractopamine (a beta-adrenergic agonist) is banned in China, and US products are tested for residues during port inspections. Even though USDA’s Export Library lists two options for meeting the ractopamine-free requirements, the Chinese government still requires a laboratory report indicating that no ractopamine was present in each lot sampled. That is, the Chinese government has yet to accept, without verification through port testing, producers and exporters who are enrolled under USDA’s AMS Process Verified Program whereby producers provide documentation that hogs were not fed ractopamine. The USDA continues to work with the Chinese government to recognize the USDA AMS program and eliminate the requirement that traders submit independent lab test reports.

In the longer run, China pork imports should continue at high levels, although a transformation is underway whereby the current domestic production structure dominated by millions of small producers is replaced by a more consolidated and modern industry. However, costs of production remain above the US and other suppliers, and imports could grow with unfettered market access.

During the absence of U.S. beef in China, Australia notably gained access to that market. However, since most Australian beef is grass-fed, it was unable to fully capture the market that U.S. producers had achieved due to meat quality demand. Historically, Japan implemented restrictive import quotas; these were phased out and replaced with a 70% tariff that was later reduced to 38.5% (Muhamma et al., 2016). However, the Japanese government enacted emergency tariff safeguards to protect local farmers. The government increased tariffs on frozen beef from 38.5% to 50% as frozen beef imports increased 17.1% during Japan’s first fiscal quarter in 2017 (United States Meat Export Federation, 2017a). By contrast, Australia faced a tariff rate of 29.9% on chilled beef and 27.2% on frozen beef (Meat and Livestock Australia, 2018).

### Bovine Spongiform Encephalopathy

In 2003, BSE was confirmed in Alberta, Canada, and in December of that year, a case was reported in the United States (Washington State). A study conducted by the Economic Research Service of the USDA looked at three markets—fresh beef, frozen beef, and frankfurters. They found no strong relationship between the news of BSE detection and altered purchasing patterns, although the first 2 wk’ beef purchases were low (Matthews et al., 2006). However, major markets for U.S. beef closed including Japan, Korea, Canada, Mexico, Egypt, and China to name a few. Beef prices in the United States dropped by 15% to 20% in the days and weeks after the announcement. Nonetheless, U.S. consumers continued to consume beef, demonstrating confidence in the U.S. beef production system. Many smaller Asian markets also opened up almost immediately. In 2014, Ecuador and Sri Lanka opened their markets to U.S. beef. Brazil reopened their market in August 2016 and China responded in June 2017.

### Beta Agonists

Beta-adrenergic receptor agonists (**BAA**), also commonly referred to as beta-agonists, are synthetic nonhormonal compounds that are commonly incorporated into the feeding regimes for beef cattle during the last few weeks prior to slaughter within the United States. While related phenethanolamines have been traditionally incorporated in human medicine to treat a range of disease (e.g., asthma, chronic obstructive pulmonary disease, bradycardia, etc.), they have also been shown to offer economic benefits and enhance meat production in beef cattle as growth promotants. Currently, ractopamine hydrochloride (Optaflexx) and zilpaterol hydrochloride (Zilmax) are the two beta-agonist that are approved by the U.S. Food and Drug Administration. Growth promotion results from the action of BAA on receptors located on the ruminant’s adipocytes and myocytes, ultimately resulting in increased rates of growth, enhanced muscle mass, improved feed efficiencies, and a down regulation of adipogenesis (Mersmann, 1998; Johnson et al., 2007). However, despite their wide use within the United States and acceptance in various countries, many countries, including European Union members and China enforce bans and restrictions on use of BAA, or presence of BAA residues in meat products even though the products may possess residues that fall within the parameters set by the Codex Alimentarius Commission (Centner et al., 2014).

## Emerging Consumer Markets

### Japan

**Figure 3.**
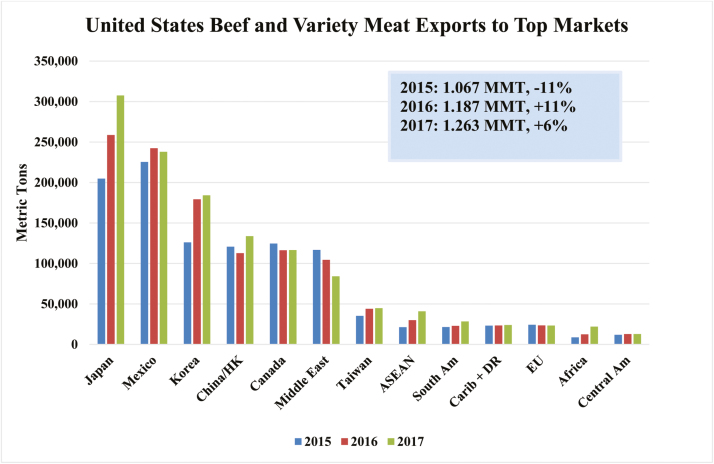
United States beef and variety meat exports to top markets (Halstrom, 2018).

Currently, Japan is ranked as the largest importer of U.S. beef. In 2017, the U.S. beef industry exported approximately 307,559 metric tons of beef and generated $1.89 billion in revenue from the Japanese market (Figure 3). This increase was a tremendous gain from 2008 where the total export volume of beef was approximately 74,119 metric tons and represents a 15.1% increase in total revenue (United States Meat Export Federation, 2017c). This current sales trend is predicted to hold constant through the year of 2018 due to the rapid growth increase in Japan’s customer–vendor services. In 2016, Japan accounted for greater than 53% of the global spending on customer–vendor services fast food items, where it is estimated that 54 supermarkets and 1,852 restaurants per 1,000 square kilometers are present in Japan (Halstrom, 2018). In addition, it is expected with the current growth of online shopping and social media influence that internet food and drink sales will rise by 53% in Japan during the period from 2016 to 2021 (Halstrom, 2018).

### Mexico

In 2017, Mexico was ranked as the second largest importer of U.S. beef (approximately 237,972 metric tons), and generated $980 billion in revenue for the U.S. beef industry, and offers to remain a significant consumer of U.S. beef products (United States Meat Exportation Federation, 2017c). This success is primarily attributed to the North American Free Trade Agreement which effectively removed all tariffs and quantitative restrictions that had previously existed between both countries in the past (United States Department of Agriculture Foreign Agricultural Service, 2017). Mexico itself offers a diverse retail market that consists of wet markets, regional retailers, and big-box stores that all focus on a wide variety of meat cuts.

### South Korea

Currently, South Korea is ranked as the third largest importer of U.S. beef and beef products and has experienced an overall sharp increase in sales from the year 2008–2017 (Figure 3). In 2017, the United States shipped 184,152 metric tons of beef to South Korea and generated about $1.2 billion in revenue with an increase in profits of 9.2% since the year 2008 (United States Meat Exportation Federation, 2017b). This current trend is expected to remain constant over the next fiscal year with the current increase in demand for ready-to-eat meals within the South Korean consumer market. The U.S. Meat Export Federation estimated that Korea’s per capita spending on ready-to-eat meals was $11.06 in 2016 and is predicted to increase by 47% to $17.00 by the end of 2021 (Halstrom, 2018). Additionally, based on per capita consumption of red meat, consumers have increased their red meat consumption by ~5% from 2005 to 2016, and this trend remains to hold constant to present day (Halstrom, 2018).

### Taiwan

According to reports released by the United States Meat Export Federation in 2017, Taiwan was ranked in seventh place for total imports of U.S. beef and beef products (United States Meat Export Federation, 2017b, Figure 3). While sales remain low compared to the other countries listed above, Taiwan has experienced steady increases between the years 2008 to 2017 with an increase in imported volumes of 17,487 metric tons, and a $282 million increase during that period. During this time, there has been a tremendous increase in demand for chilled, frozen, and shelf-stable meat products, and is predicted to increase by 23% by 2021 (Halstrom, 2018). In addition, it has been reported that in 2017 between January and August Taiwan’s chilled beef imports from the United States has increased by 16%, and to date, the United States holds approximately 70% of the market share in imported chilled beef (Halstrom, 2018).

## What Does the Future Hold?

The global population is expected to increase to 9.2 billion or more by 2050, with the majority of the population growth expected to take place in developing countries. The rural population is likely to decline in the next decade as urban areas will account for 70% of the world population in 2050 (Food and Agriculture Organization, 2009). As urbanization is accelerating, the overall global economic growth is expected to be about 2.9% annually. The global population will continue to face economic deprivation and malnutrition. As the population is rapidly growing, food production is expected to increase by 70%, to feed this expanding population. Therefore, production of essential commodities will also have to rise. Meat production, for instance, will have to grow by over 200 million tons. Beef, veal, pork, and poultry per capita consumption has increased 3% annually in developing countries since the mid-1990s. During this time, growth in developed countries has only been about 0.4% (Westcott and Hansen, 2015).

By 2020, the middle class is projected to become the majority of the global population. As the global population has increased disposable income, there will be an upgraded demand for food (Halstrom, 2018). These new upgrades present new opportunities for the agricultural community and meat industries. The United States is recognized for offering safe, affordable food and will be a significant player in providing animal protein to meet the growing demand at an efficient rate. Innovations and technologies today allow for agriculture organizations, business arrangements, and production practices to enable more production with less input. For instance, land use has declined over time where land used in agriculture dropped from 54% to 51% of total U.S. land area from 1982 to 2007 (Nickerson and Borchers, 2012). Land use for beef production has declined by 34% since 1977, and the total amount of water used in each pound of beef production has dropped by 14% (Ishmael, 2013).

New export opportunities are available through mainland China as they recently re-opened their market to the U.S. beef in June 2017. After a 14-yr ban because of BSE, the amount of beef exports increased each month during 2017. The U.S. Department of Agriculture’s Foreign Agricultural Service has projected China will import 2.26 billion pounds of beef in 2018. However, on April 4, 2018, the Chinese government proposed a tariff of 25% on China’s imports of agricultural and food products from the United States (USDA GAIN, 2018). This tariff can impose a risk on business relationships and opportunities for further growth.

**Figure 4.**
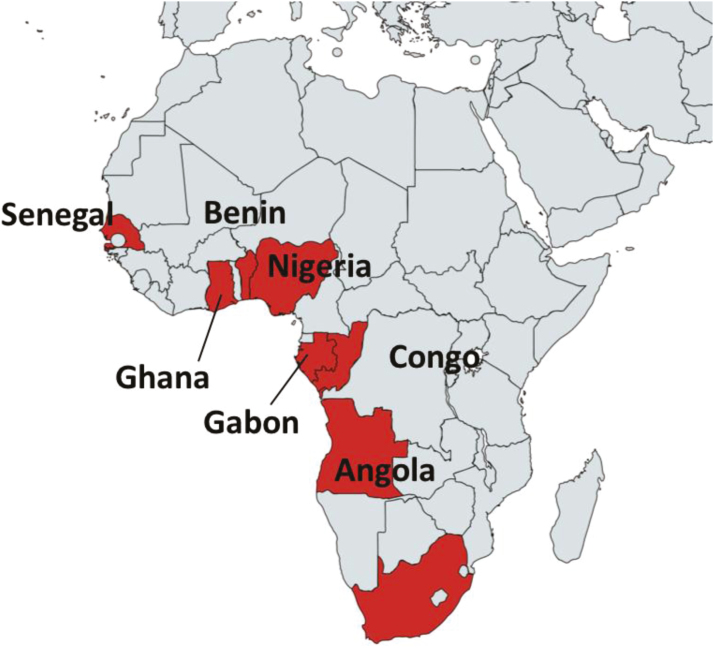
New opportunities for export of beef to Africa (Halstrom, 2018).

South Africa reopened its market to U.S. beef in 2016 and emerged as a promising market with new opportunities especially for beef variety meat (Figure 4). The United States is the second largest beef variety meat supplier in 2017, capturing 24% of the imported beef and beef variety markets to South Africa (U.S. Meat Export Federation, 2017b). The re-opening of South Africa has allowed for beef livers to be delivered in diverse areas (Halstrom, 2018). Egypt remains the largest market for U.S. beef livers.

## Conclusions

In closing, due to its high-quality standards, established safety protocols, and well-designed infrastructure, the U.S. beef industry has managed to maintain a strong and an ever-increasing international presence among its competitors during recent years. Coupled with its continuous growth in existing international markets (e.g., Japan, Mexico, South Korea, etc.) during the last decade, and emerging markets (e.g., China and Africa) with high demand, the U.S. beef industries have opportunities for diversifying and expanding retail for beef and offal products. However, while the future for U.S. beef exports appears optimistic and strong, the industry will continue to face barriers to trade and must remain innovative in order to produce sufficient volume and quality beef to satisfy its existing and expanding markets.

## About the Authors

**KathrynAnn Hunter Fields** majored in food and resource economics at the University of Florida, earning her bachelor’s degree in 2013 and master’s in 2015. She is a 2018 graduate of the Bush School of Government and Public Service at Texas A&M University, completing a master’s degree with an emphasis in Agriculture Policy. Katie completed an internship with the National Cattlemen’s Beef Association in Washington, D.C., in Spring 2015, and was one of six ASAS Science Policy interns in Summer 2017. In her spare time, Katie enjoys travelling and assisting her father on the family ranch in Florida and volunteering in the Bryan-College Station community.

**Dustin Aaron Therrien** received his bachelor’s degree in cell and molecular biology in 2013, and master of science in 2015, with emphasis on virology of the equine infectious anemia virus, from Stephen F. Austin State University. He is currently a doctoral student in Animal Science at Texas A&M University with research focus on applying next generation sequencing technologies within the U.S. food and agricultural industries for improved meat quality of beef and beef products, and with application to food safety.

**Dan Halstrom,** USMEF President and CEO. Dan was named President of USMEF effective September 1, 2017 and President & CEO effective December1, 2017. Dan Joined USMEF in 2010 after 27 years in the meat industry with Swift & Company originally based in Chicago, and now doing business as JBS based in Greeley Colorado. Dan was responsible for the global marketing for USMEF including coordination of programs in 18 regions around the world facilitating the marketing of US Beef, Pork, and Lamb. In addition, Dan oversees the industry relations and membership activities for USMEF and their nine sectors of membership. Dan began his role at Swift as a management trainee, spending several years at the plant level, before moving into commercial sales and eventually into International Sales where he spent most of his career. As senior vice president of the International Division for Swift/JBS (2000–2010), he oversaw global beef and pork operations for the world’s largest red meat company. Dan was also an active member of USMEF for many years, including various volunteer positions including the officer role with USMEF from 2005 to 2007 which culminated as Chairman of USMEF in 2008. A graduate of the University of Iowa, Dan was born and raised near Cherokee, Iowa, on a 1,000-acre grain farm that also had a 3,000-head cattle backgrounding operation.

**Joel Haggard**, Senior Vice President - Asia Pacific Region, U.S. Meat Export Federation. Mr. Haggard joined USMEF in 1988 as a regional marketing consultant and later became director of the USMEF-Hong Kong office when that office opened in 1989. Joel Haggard returned to the Asia-Pacific to serve as vice president-Asia Pacific for the U.S. Meat Export Federation in March 1994, after several years in the USMEF-Denver office. Stationed in the USMEF-Hong Kong office, Mr. Haggard oversees USMEF market development strategies and programs for U.S. red meat products in the region. Before joining USMEF, Mr. Haggard worked for the USDA Foreign Agricultural Service for 5 years. During that time, he served as the agricultural trade officer for the U.S. Embassy in Beijing, China, where he supervised all marketing activities involving U.S. agricultural trade with the People’s Republic of China. Prior to that, Mr. Haggard worked as grain analyst for USDA’s Foreign Agriculture Service in Washington, DC, analyzing and forecasting international trade patterns. Mr. Haggard has a Bachelor of Science Degree in Political Economy of Natural Resources from the University of California, Berkeley, and a Master of Science Degree in Agricultural Economics from the University of Wisconsin. He speaks both Mandarin and Cantonese.

**Paul Clayton** is Senior Vice President of Export Service of the US Meat Export Federation (USMEF) and administers the Export Service Programs for exporting members of the USMEF. These programs include market access solutions in foreign countries, industry relations and various member services including research, technical, and regulatory services. Mr. Clayton has over 30 years of experience in the meat industry. He received Bachelor and Master Degrees in Animal Science from Colorado State University. He was employed by Monfort/ConAgra for over 17 years where he held several technical, managerial, and executive positions. Mr. Clayton was also Vice President of Technical Services for the processed meat division of J.R. Simplot. He has been with USMEF since 2000. Mr. Clayton was a member the National Advisory Committee on Microbiological Criteria for Foods, Leader for development of the Generic HACCP Model for Beef Slaughter for the International Meat and Poultry HACCP Alliance and Chairman of the Colorado Governor’s Task Force on Food Safety. Mr. Clayton was co-recipient of the Edison Award for Best New Product Introduced in 1991 and also is co-inventor on seven U.S. Patents. Mr. Clayton is active in several professional affiliations. He currently participates as a member of several committees for, NAMI, AMSA, NCBA, and NPB and is a member of the NWSS International Committee and the Executive Committee for the International Stockman’s Education Foundation. Mr. Clayton and his wife Cheryl reside in Parker Colorado.
